# There Goes the (Gene Expression) Neighbourhood Theory

**DOI:** 10.1371/journal.pbio.1001002

**Published:** 2010-11-30

**Authors:** Rachel Jones

**Affiliations:** Freelance Science Writer and Editor, Welwyn, Hertfordshire, United Kingdom

**Figure:**
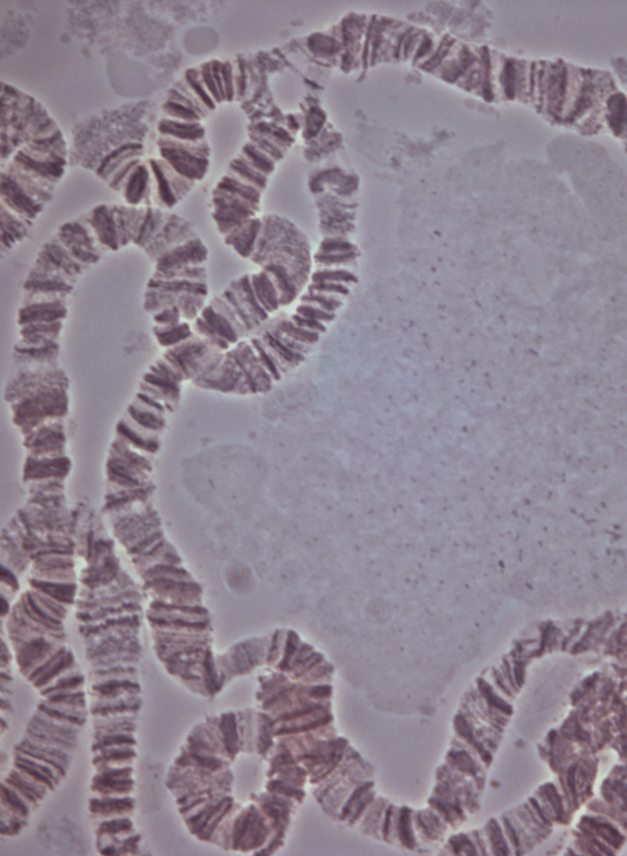
A polytene chromosome spread showing a designer inversion disrupting a gene expression neighbourhood in the *Drosophila* genome.

The production of proteins from their respective genes is a tightly regulated process that helps cells conserve energy while ensuring they have the raw materials needed to perform basic life processes. The expression of any particular gene can be controlled by various mechanisms, including the actions of transcription factors and repressors, and epigenetic factors such as DNA methylation or chromatin remodelling. In addition, many eukaryotic genomes, from plants to humans, appear to have another regulatory trick at their disposal: “gene expression neighbourhoods”—areas in which genes that are close together tend to have similar expression profiles. Many researchers have proposed that the organization of these neighbourhoods might be important for the coordinated expression of the genes that reside within them.

In a new study, Meadows et al. have tested this theory in the fruitfly *Drosophila* by disrupting several gene expression neighbourhoods that contain testis-specific genes. Their results undercut the notion that gene expression neighbourhoods may play a regulatory role.

Several gene expression neighbourhoods have been shown to be evolutionarily conserved among species, raising the possibility that there is some selective advantage to keeping these genes together in the genome. In *Drosophila*, at least 20% of all genes seem to be clustered into neighbourhoods, and one study concluded that around 45% of genes that are expressed exclusively in the testes were found in testis-specific gene expression neighbourhoods. Might this clustering into neighbourhoods somehow facilitate the coordinated expression of genes that have similar expression profiles? To find out, Meadows et al. generated targeted chromosomal inversions in the fly genome to split up specific gene expression neighbourhoods populated by testis-specific genes. They then used microarray analysis to compare the expression of the genes found in the affected neighbourhoods in flies carrying the inversions with otherwise genetically identical flies that did not carry the inversions.

Perhaps surprisingly, the authors found no significant differences in gene expression between the flies with the inversions and those without. Even though the testis-specific genes no longer occupied the same neighbourhood (and thus were no longer clustered in the same way as in the wild-type genome), their expression patterns were normal. This finding was consistent whether the authors measured gene expression in the whole flies or in just the testes.

They also generated flies with an inversion in an embryo expressed gene neighbourhood and again found no significant effect on gene expression, suggesting that the lack of effect of the inversions was not confined to sexually dimorphic genes or to the testis-specific genes themselves.

One effect of gene clustering might be to ensure that genes are physically close together in the nucleus, perhaps bringing them under the influence of common factors that might affect their transcription. To investigate whether the clustered genes were physically separated in the nucleus by the chromosomal inversions, the authors used fluorescent markers to measure the distance between the genes. Genes that came from the same neighbourhood, but which were split apart by an inversion, were significantly further apart in the nucleus than the same genes in wild-type flies, which suggests that co-localization in the nucleus did not overcome the effects of the inversions.

Meadows et al. have shown that, at least in *Drosophila* and at least for certain clusters of genes, the existence of gene expression neighbourhoods is not necessary for the correct and coordinated expression of genes that have the same expression profiles. These findings suggest that one shouldn't assume that genes inhabit the same neighbourhood for a reason. Why these clusters have weathered the passage of evolutionary eons, however, remains an open question. It may well be, the authors suggest, that the evolutionary advantage of neighbourhoods might lie in more subtle effects, which await the creation of more powerful tools to reveal their purpose.

**Meadows LA, Chan YS, Roote J, Russell S (2010) Neighbourhood Continuity Is Not Required for Correct Testis Gene Expression in *Drosophila*. doi:10.1371/journal.pbio.1000552**

